# Characterization of microbial communities in the chicken oviduct and the origin of chicken embryo gut microbiota

**DOI:** 10.1038/s41598-019-43280-w

**Published:** 2019-05-02

**Authors:** Sangwon Lee, Tae-Min La, Hong-Jae Lee, In-Soo Choi, Chang-Seon Song, Seung-Yong Park, Joong-Bok Lee, Sang-Won Lee

**Affiliations:** College of Veterinary Medicine, Konkuk University, 120 Neungdong-ro, Gwangjin-gu, Seoul 05029 Republic of Korea

**Keywords:** Metagenomics, Microbiome

## Abstract

The transferred microbiota from mother to baby constitutes the initial infant gastrointestinal microbiota and has an important influence on the development and health of infants in human. However, the reproductive tract microbiota of avian species and its inheritance have rarely been studied. We aimed to characterize the microbial community in the chicken reproductive tract and determine the origin of the chicken embryo gut microbiota. Microbiota in four different portions of chicken oviduct were determined using 16S rRNA metagenomic approach with the IonTorrent platform. Additionally, we analyzed the mother hen’s magnum and cloaca, descendent egg, and embryo gut microbiota. The microbial composition and relative abundance of bacterial genera were stable throughout the entire chicken reproductive tract, without significant differences between the different parts of the oviduct. The chicken reproductive tract showed a relatively high abundance of *Lactobacillus* species. The number of bacterial species in the chicken reproductive tract significantly increased following sexual maturation. Core genera analysis detected 21 of common genera in the maternal magnum and cloaca, descendent egg shell, egg white, and embryo gut. Some elements of the maternal oviduct microbiota appear to be transferred to the embryo through the egg white and constitute most of the embryo gut bacterial population.

## Introduction

The presence and composition of normal microflora in the female reproductive tract have been previously studied in humans, non-human primates, and other mammals^1–4^. The vaginal microbiota of human changes to a *Lactobacillus* species- or other lactic acid bacteria-dominant community with sexual maturity and becomes less diverse and enriched for *Lactobacillus* species during pregnancy^5–7^. These changes in the vaginal microbiota are known to maintain a low pH and provide bactericidal substances to prevent pathogen infection and growth. In addition, the microflora of the reproductive tract is transferred to babies during natural delivery, constituting the initial gastrointestinal microbiota of infants^8,9^, which has an important influence on infant development and health^10^. Moreover, human studies have demonstrated the presence of microflora in the upper reproductive tract, with higher species diversity and lower frequency of *Lactobacillus* compared to the vaginal microbiota^11,12^. Although the core microbial features and roles of the microbiota in the upper reproductive tract have not been precisely determined, disruption of its normal composition is related to preterm birth and uterine-related diseases^13,14^. It has also been suggested that the bacterial community initially colonizes the gut of the fetus through the placenta in utero^15,16^.

Studies examining the microbiota of the reproductive tract lining, especially the vagina, have also been conducted in non-human primates and livestock animals^3,4,17^. However, in these species, the vagina has a unique microbiota with low levels of lactobacilli and the pH of the vagina is near neutral, except in the chimpanzee and some baboon species^3,4,18,19^. In addition, the roles of the vaginal microbiota in these animal species have not been fully elucidated.

In contrast, the microflora in the oviduct of avian species has rarely been studied, although colonization of the reproductive tract of poultry by bacterial pathogens, including S*almonella* species, and vertical transmission of these pathogens to offspring have been described in previous studies^20–24^. Currently, the gut microbiota of newly hatched chicks is thought to originate from fecal or environmental contaminants attached to egg shells^25^. A recent study has suggested microbiota inheritance from mother hens to chicks based on a comparison of their fecal and gastrointestinal microflora; maternal hen-derived microbiota transmission to the embryo via the egg formation process in the oviduct has also been suggested^26^. However, to date, the association between the mother hen’s oviduct, feces, and egg shell microbiota and that of its offspring have not been studied simultaneously using a metagenomic approach with next-generation sequencing (NGS) technology. In this study, we aimed to investigate the microbial community throughout the chicken oviduct and its variation following sexual maturity, as well as the correlation between the maternal oviduct and chicken embryo microbiota.

## Results

We analyzed the microbial communities in a total of 92 samples originating from commercial and specific pathogen-free (SPF) hens, their descendant eggs, and embryos. Following sequence processing and sample filtering, we sorted 2,843,017 quality reads (mean 30,902.35 ± 19,258.25) into 2,255 (165.57 ± 92.43) operational taxonomic units (OTUs) using a sequence identity cutoff of 99%. Negative control resulted 32 raw sequences, all of them were filtered out during sequence processing step. All samples were rarified to 5,000 reads.

### Microbiota of the chicken reproductive tract

**Figure 1:**
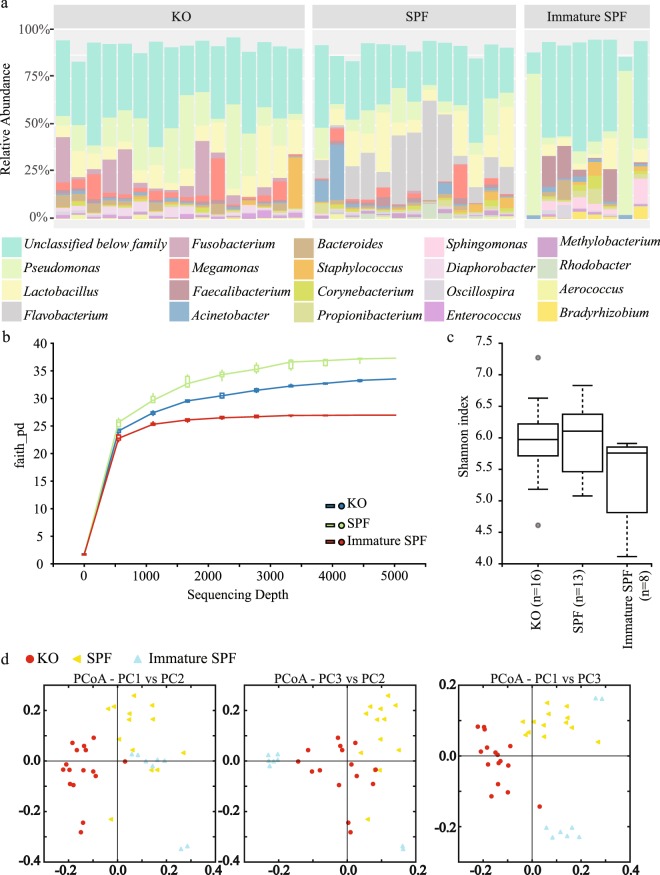
Comparison of microbial population in the oviducts of Korean commercial and SPF chickens. (**a**) Taxonomic diversity plot showing the relative abundance of taxa at the genus level in each oviduct sample. Twenty of the most abundant taxa are displayed and sorted by different colors. The relative abundance of each sample was calculated based on the OTU table rarefied to 5000 reads per sample. (**b**) Faith’s phylogenetic diversity (PD) rarefaction curve comparing oviduct groups. (**c**) Shannon’s diversity index of each oviduct group. Boxplots show the quartiles, median, and extremities of the values. (**d**) 2D PCoA plot based on unweighted UniFrac distance matrix. Each circle or triangle represents an oviduct sample. KO: oviduct samples including infundibulum, isthmus, magnum and uterus from Korean commercial breed chickens; SPF: magnum samples from SPF breed chickens; Immature SPF: magnum samples from immature SPF breed chickens.

We collected 16 samples from different sections (infundibulum, magnum, isthmus, and uterus) of four oviducts of the egg-laying Korean commercial breed hens to analyze the composition of microbial communities in the chicken reproductive tract. Pairwise permutational multivariate analysis of variance (PERMANOVA) was conducted based on an unweighted UniFrac matrix. No significant differences in microbial composition along the chicken reproductive tract were observed (Table 1 and Supplementary File 2). Over 99% of the reproductive tract microbiota of the Korean commercial breed hens was comprised of *Firmicutes* (43.65% ± 22.65%), *Proteobacteria* (39.44% ± 22.07%), *Fusobacteria* (8.06% ± 10.12%), *Bacteroidetes* (5.47% ± 3.57%), and *Actinobacteria* (2.86% ± 1.84%) at the phylum level (Fig. S1 in Supplementary File 1). At the genus level, taxa unclassified below family level were the most abundant (38.58 ± 8.09%) followed by *Pseudomonas* (17.30 ± 12.69%), *Lactobacillus* (11.13 ± 7.47%), *Fusobacterium* (7.76 ± 9.89%), *Megamonas* (4.93 ± 5.70%), *Bacteroides* (2.37 ± 2.09%), *Staphylococcus* (2.15 ± 6.59%), *Diaphorobacter* (2.14 ± 1.71%), and *Enterococcus* (1.29 ± 0.89%) (Fig. 1a). At the species level, the lactobacilli consisted of *L*. *salivarius* (58.73%), unclassified *Lactobacillus* species (36.11%), *L*. *reuteri* (2.11%), *L*. *delbrueckii* (1.09%), and four minor (<1%) *Lactobacillus* species: *L*. *agilis*, *L*. *helveticus*, *L*. *iners*, and *L*. *vaginalis*.

**Table 1:** Pairwise PERMANOVA statistic based on unweighted UniFrac distance matrix.

Group 1	Group 2	pseudo-F	p-value	q-value
Infundibulum	Isthmus	1.166965	0.281	0.529
	Magnum	0.91175	0.509	0.529
	Uterus	1.110122	0.435	0.529
Isthmus	Magnum	0.893045	0.529	0.529
	Uterus	1.268723	0.364	0.529
Magnum	Uterus	1.225458	0.386	0.529

*p-values were calculated based on 999 permutation tests.

To examine variations in the chicken reproductive tract microbiota according to breed, we collected 16 magnum samples from 34 weeks-old SPF hens. Of them, we used 13 data samples that passed quality filtering. At the phylum level, Firmicutes (32.48% ± 21.36%), Proteobacteria (31.40% ± 12.73%), Bacteroidetes (25.20% ± 15.02%), and Actinobacteria (10.09% ± 5.39%) accounted for >99% of the microbiota of magnum in SPF breed hen. Fusobacteria only accounted for 0.22% ± 0.42% of the population, while it accounted 6.902% of the microbiota from the Korean commercial breed (Fig. S1 in Supplementary File 1). At the genus level, taxa unclassified below family level were the most abundant (30.09 ± 7.13%), followed by Flavobacterium (19.76 ± 15.43%), Lactobacillus (13.49 ± 10.53%), Pseudomonas (8.87 ± 5.99%), Acinetobacter (4.15 ± 8.27%), Megamonas (3.06 ± 4.88%), Rhodobacter (1.62 ± 2.13%), Faecalibacterium (1.48 ± 1.39%), Staphylococcus (1.41 ± 1.83%), Propionibacterium (1.13 ± 1.66%), Corynebacterium (1.10 ± 0.88%), Methylobacterium (1.05 ± 0.84%), and Bacteroides (1.02 ± 0.76%) (Fig. 1a). In contrast to the Korean commercial breed, SPF chickens had a high level of Flavobacterium in the magnum. At the species level, the lactobacilli were comprised of unclassified Lactobacillus species (61.75%), L. helveticus (12.43%), L. salivarius (9.22%), L. reuteri (8.28%), L. vaginalis (5.99%), L. pontis (1.88%), and two minor (<1%) Lactobacillus species: L. agilis and L. coleohominis. The phylogenetic diversity (PD) values, representing species richness, and the Shannon index, representing alpha diversity, were similar between the two chicken breeds (Fig. 1b,c and Table 2). A 2D principal coordinates analysis (PCoA) plot constructed from the unweighted UniFrac matrix showed the microbiota in the mature hen oviduct were separately grouped by breed (Fig. 1d).

**Table 2:** Pairwise Kruskal-Wallis test for phylogenetic diversity of oviduct* groups.

Group1	Group2	H	p-value	q-value
KO	SPF	0.001923	0.965022	0.965022
	Immature SPF	8.640000	0.003289***	0.009866
SPF	Immature SPF	6.062937	0.013805**	0.020707

*KO group includes infundibulum, isthmus, magnum and uterus samples of Korean commercial breed hens, and SPF group includes magnum samples of SPF breed hens.

** indicates the p-value < 0.05 and ***indicates the p-value < 0.01.

In order to analyze the effect of sexual maturity on the microbiota in chicken reproductive tract, we collected eight magnum samples from 23 weeks-old immature SPF hens. At the phylum level, *Proteobacteria* (56.56%), *Firmicutes* (26.00%), *Actinobacteria* (13.37%), and *Bacteroidetes* (3.53%) accounted for >99% of the microbiota in the immature hen magnum (Fig. S1 in Supplementary File 1). At the genus level, taxa unclassified below family level were the most abundant (40.60 ± 19.89%) followed by *Pseudomonas* (22.47 ± 33.17%), *Faecalibacterium* (7.63 ± 5.58%), *Lactobacillus* (4.82 ± 5.36%), *Sphingomonas* (3.96 ± 4.24%), *Propionibacterium* (2.15 ± 2.68%), *Janthinobacterium* (2.05 ± 3.83%), *Bradyrhizobium* (1.91 ± 2.32%), *Bacteroides* (1.83 ± 3.57%), *Acinetobacter* (1.79 ± 0.75%), *Corynebacterium* (1.77 ± 2.34%), *Oscillospira* (1.43 ± 2.39%), and *Staphylococcus* (1.39 ± 2.50%) (Fig. 1a).

The Faith PD and Shannon index value of the immature hen group were significantly lower than those of mature hens and showed large individual variance (Fig. 1b,c). In the 2D PCoA plot, the immature hen group was separated from the mature hen groups belonging to two different chicken breeds (Fig. 1d).

**Figure 2:**
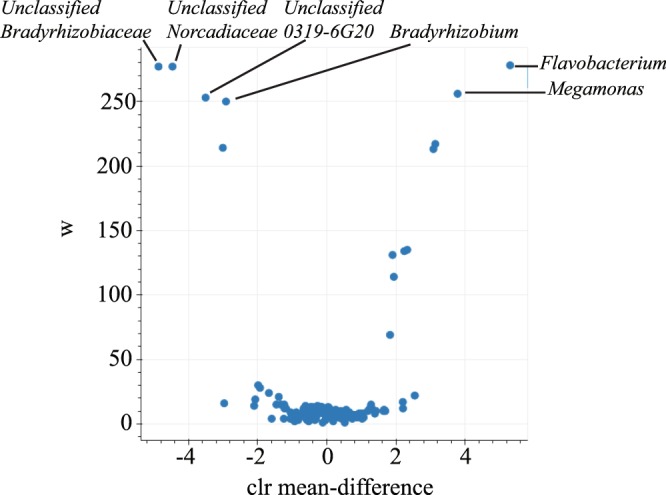
ANCOM differential abundance volcano plot. For ANCOM analysis, the clr (centered log ratio) transformed OTU table at the genus level that was modified to adjust 0 values to 1 was used. The W value represents the number of times of the null-hypothesis (the average abundance of a given species in a group is equal to that in the other group) was rejected for a given species. The x-axis value represents the clr transformed mean difference in abundance of a given species between the mature and immature hen magnum groups. A positive x-axis means a species is abundant in mature chicken magnum and a negative x-axis value means a species is abundant in immature chicken magnum. Only species with reject null-hypothesis >95% are labelled.

As the PD value and Shannon index were significantly lower in immature hen magnum than in mature hen magnum and other parts of oviduct (Fig. 1), we performed a random forest test and analysis of composition of microbiome (ANCOM)^27^ to identify key genera discriminating the microbiota of mature hens from those of immature hens. Of the 281 observed taxa at the genus level, unclassified *Nocardiaceae* (W = 277), *Bradyrhizobiaceae* (W = 277), and *0319-6G20* (W = 250) and genus *Flavobacterium* (W = 278), *Megamonas* (W = 256), and *Bradyrhizobium* (W = 253) showed a significant difference (p < 0.05) in abundance between microbiota in the mature and immature SPF hen magnum. Unclassified *Nocardiaceae*, *Bradyrhizobiaceae*, and *0319-6G20* and genus *Bradyrhizobium* were relatively abundant in the immature hen magnum and genus *Flavobacterium* and *Megamonas* were relatively abundant in mature hen magnum (Fig. 2).

A random forest test with 500 decision trees was performed to identify important genera for predicting oviduct maturity; samples were classified into each category with an overall model accuracy of 1 and baseline accuracy of 0.6. The genera increasing the prediction error rate over 5% are listed in Table 3. The core genera, classified below family level, present in all samples of each group are listed in Table 4. The core genera consisted of 19 and 11 genera classified below family level in the Korean commercial breed chickens and in SPF chickens, respectively. Of these, unclassified *Caulobacteraceae*, *Lachnospiraceae*, and *Enterobacteriaceae* and genus *Pseudomonas*, *Lactobacillus*, *Megamonas*, *Bacteroides*, and *Oscillospira* were found in all mature hen magnum and other parts of oviduct of Korean commercial breed hen. In immature hen magnums, only *Pseudomonas* and *Acinetobacter* constituted the core genera.

**Table 3:** Important genera for predicting chicken maturity.

Genus	Importance*
Genus *Rhodobacter*	8.119173
Genus *Flavobacterium*	7.715198
Class *Betaproteobacteria*	7.212965
Genus *Megamonas*	5.9891

*Importance represents an increase in error rate (%) when the genus is excluded from maturity prediction analysis.

**Table 4:** Core genera* of the reproductive tract of Korean commercial breed and SPF chickens.

Group	Family	Genus
KO	*Pseudomonadaceae*	*Pseudomonas*
	*Lactobacillaceae*	*Lactobacillus*
	*Veillonellaceae*	*Megamonas*
	*Bacteroidaceae*	*Bacteroides*
	*Enterococcaceae*	*Enterococcus*
	*Ruminococcaceae*	*Oscillospira*
	*Comamonadaceae*	*Diaphorobacter*
	*Ruminococcaceae*	*Faecalibacterium*
	*Corynebacteriaceae*	*Corynebacterium*
	*Weeksellaceae*	*Cloacibacterium*
	*Pasteurellaceae*	*Gallibacterium*
	*Xanthomonadaceae*	*Stenotrophomonas*
	*Comamonadaceae*	*Tepidimonas*
	*Lachnospiraceae*	Unclassified
	*Comamonadaceae*	Unclassified
	*Caulobacteraceae*	Unclassified
	*Enterobacteriaceae*	Unclassified
	*Clostridiaceae*	Unclassified
	*Bradyrhizobiaceae*	Unclassified
SPF	*Pseudomonadaceae*	*Pseudomonas*
	*Lactobacillaceae*	*Lactobacillus*
	*Veillonellaceae*	*Megamonas*
	*Bacteroidaceae*	*Bacteroides*
	*Ruminococcaceae*	*Oscillospira*
	*Moraxellaceae*	*Acinetobacter*
	*Flavobacteriaceae*	*Flavobacterium*
	*Rhodobacteraceae*	*Rhodobacter*
	*Caulobacteraceae*	Unclassified
	*Enterobacteriaceae*	Unclassified
	*Lachonospiraceae*	Unclassified
Immature	*Pseudomonadaceae*	*Pseudomonas*
	*Moraxellaceae*	*Acinetobacter*

*The genera detected in all samples in each group were considered as the core genera. Species unclassified below family level were excluded.

### Origin of chicken embryo gut microbiota

To compare the microbial communities of mother hens and descendent embryos in detail, we used a total 55 of samples from the cloaca (n = 14) of mother hens, egg shells (n = 13), egg white (n = 14) from descendent eggs, and the cecum (n = 14) of descendent chicken embryos of the SPF hens used for magnum sampling.

**Figure 3:**
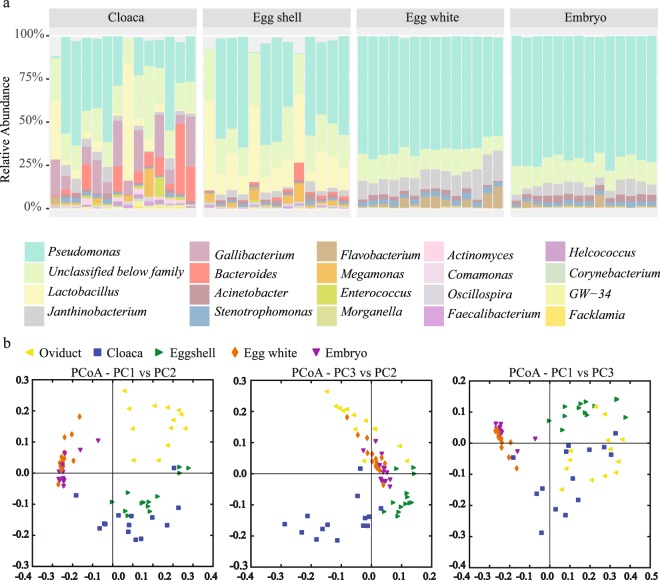
Correlation between mother hen, descendent egg, and embryo microbiota. (**a**) Taxa relative abundance in each sample at the genus level. Each bar represents a sample and samples were separated by their sampling location indicated above the bar. Twenty of the most abundant genera across all samples were included in the plot. (**b**) PCoA plot based on an unweighted UniFrac distance matrix. Samples were sorted by their sampling location indicated at the top left of the figure. Oviduct, SPF hen magnum; Cloaca, SPF hen cloaca; Egg shell, egg shell surface of eggs laid by SPF hens; Egg white, egg white of eggs laid by SPF hens; Embryo, cecum of 18-day-old chicken embryos laid by SPF hens.

The relative taxa abundance plots at the genus level showed that egg white and embryo cecum had a similar microbial composition with lower diversity than mother hen cloaca or magnum (Figs 3a, S2 and S3 in Supplementary File 1). In the PCoA plot, chicken embryo ceca samples co-localized with the egg white samples (Fig. 3b).

**Figure 4:**
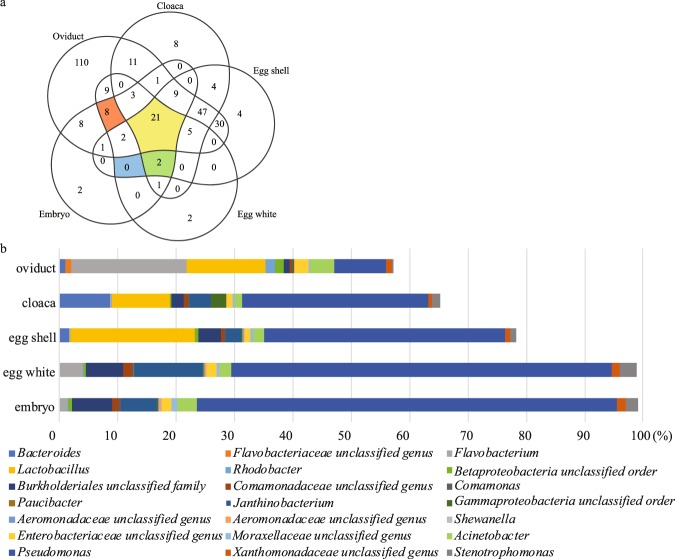
Shared and unique phylotypes at the genus level in the mother hens, egg and embryo. (**a**) Venn diagram showing the number of phylotypes at the genus level in the maternal magnum and cloaca, descendent egg shell, egg white, and embryo. Phylotypes observed in any samples in a group were counted. Red-colored intersection, phylotypes found only in magnum, egg white and embryo; blue-colored intersection, phylotypes found only in egg shell, egg white, and embryo; green-colored intersection, phylotypes found only in cloaca, egg shell, egg white, and embryo; yellow-colored intersection, phylotypes found in all groups. (**b**) Mean values of relative abundance of the 21 common phylotypes in each group presented as % value.

The distance between each sample group based on unweighted UniFrac distance matrix was calculated by analysis of similarities (ANOSIM) with 999 permutations. The result revealed that egg white and embryo cecum were very similar to each other (R = 0.12, p = 0.001). However, egg white and embryo combined groups were too different from other groups to be compared; the egg shell (R = 0.97, p = 0.001), cloaca (R = 0.91, p = 0.001) and magnum (R = 0.98, p = 0.001). To elucidate the contribution of the microbial community in the mother hen’s magnum and cloaca, or egg shell to the egg white or embryo gut microbiota, SourceTracker^28^ analysis was performed in all directions. In this analysis, egg white and embryo samples were grouped into same group for better sink prediction. The results revealed that the egg shell microbiota contributed approximately 63% of the embryo cecum and egg white microbiota. When egg shell was assigned as the sink, the magnum and cloaca microbiota contributed 28% and 17%, respectively, of the egg shell microbiota. Unknown source contribution accounted over 50% in cloaca and magnum (Table 5). Figure 4a shows the number of shared phylotypes at the genus level among the descendent embryo, egg white, egg shell, cloaca, and magnum microbiota. Of the 288 of phylotypes, 21 were observed in all groups. The relative abundance of the 21 common phylotypes at the genus level in each group is presented in Fig. 4b and Supplementary File 2. These taxa account for 99, 98, 78, 65, and 47% of the embryo, egg white, shell, cloaca, and magnum bacterial population, respectively. Eight phylotypes were observed only in magnum, egg white, and embryo gut, while two phylotypes were observed in all groups except magnum. No phylotypes were observed only in the embryo, egg white, or egg shell.

**Table 5:** The mean contribution value of each source to each sink.

Sink	Egg white and embryo	Cloaca	Egg shell	Magnum	Unknown	Mean SD*
Egg white and embryo	—	0.31746	0.633795	0.032087	0.016658	0.008804
Cloaca	0.378369	—	0.107249	0.009446	0.504937	0.06901
Egg shell	0.443322	0.174962	—	0.28268	0.099037	0.011953
Magnum	0.030991	0.003092	0.195612	—	0.770305	0.003691

*The mean value of the standard deviation of each fractional contribution.

## Discussion

The human vaginal microbiota and its roles in the mother and infants have been described in several previous studies^29–33^. The vaginal microbiota of healthy reproductive women is dominated by *Lactobacillus* species^1^. In contrast, in other mammalian animals, *Lactobacillus* species make up a very small fraction of the total vaginal microbial population and alpha diversity indexes are notably higher than in the human vaginal microbiota, suggesting that *Lactobacillus* dominance and low species diversity are unique to the human vagina^2–4,19^. In addition, the dominant bacterial genera in the reproductive tract of these non-human animal species are different from one another^2–4,19^. However, the microbiota of non-mammalian animals, including avian species and reptiles, has rarely been studied. Our study aimed to characterize the microbiota of the chicken reproductive tract and determine the influence of sexual maturity on microbial population. Unlike in humans, where the vaginal and upper reproductive tract microbiota are significantly different, the mature hen oviduct microbiota did not show significant differences along the tract. However, in order to ensure this result, large number of samples will be needed in further study. The upper reproductive tract microbiota more closely resembled the human upper reproductive tract microbiota than the lower reproductive tract microbiota with regards to the relatively low frequency of *Lactobacillus* and high species diversity^11,12^. The chicken reproductive tract microbiota was predominantly comprised of *Firmicutes*, *Proteobacteria*, *Bacteroidetes*, and *Actinobacteri*a. At the genus level, *Pseudomonas*, *Lactobacillus*, *Megamonas*, and *Bacteroides* were most abundant and represented the core bacterial genera in the mature hen reproductive tract. These major genera were apparently different from those present in the reproductive tract microbiota of other animals^3,4,19^. Lactobacilli were detected in all mature hen oviduct samples; although their relative abundance (12.19% ± 8.88%) was lower than in the mature human vaginal microbiota^1^, it was higher than in other mammalian species^3,4,19,34^. While human infants delivered naturally receive lactobacilli from the microbiota present in the mothers’ vagina, the chicken embryo gut had only a very low number of lactobacilli. These results suggest that the lactobacilli in the chicken oviduct do not transfer to offspring and have a different role in the chicken and human reproductive tract^9^.

The reasons underlying the prevalence and dominance of *Pseudomonas* species in the reproductive tract and embryo are uncertain. Most *Pseudomonas* spp. detected in this study were unclassified below the genus level (86.8% of reads assigned as *Pseudomonas*) and 12.4% of these were *P*. *veronii*, a member of the *P*. *fluorescens* group^35^. Though the role of *P*. *veronii* in animals is unknown, based on recent studies, it may be involved in protecting the reproductive tract against bacterial pathogens or parasite infection^36–38^.

*Megamona*s spp., which were not detected in the immature chicken oviduct, were detected in all mature hen oviduct samples. These species were also detected in most cloaca and egg shell samples. However, this genus was not detected in any of the egg white and embryo samples, except for one embryo sample with 0.1% abundance. These findings imply that although *Megamonas* species present in the mature hen reproductive tract, they cannot pass through the barrier between the egg shell surface and egg white. Similar to *Megamonas* species, some bacterial species originating from the mother hen oviduct were unable to pass the egg shell-shell membrane barrier resulting in low species diversity in the egg white and embryo ceca. These species could play a role in protecting the reproductive tract and eggs against infection rather than constituting part of the offspring microbiota. Therefore, the interaction between hen and these species should be studied to understand the function of the reproductive tract microbiota of hen.

Microbiota in magnum samples between Korean commercial breed and SPF chicken breed showed taxonomic difference in this study. However, we used only four oviducts from Korean commercial breed chickens. This was not enough number to confirm characteristics of microbial community in those samples. To understand the relationship between reproductive tract microbiota of hen and factors influencing on it, adequate number of samples will be needed in further study. Also, though we only took account of breed as a factor that influence composition of microbiota in chicken reproductive tract. However, the other important factors, including diet, immune status, and environment should have been considered.

Based on results of this study, it appears that the chicken embryo obtains its intestinal microbiota from the egg white prior to birth. The embryo and egg white samples were grouped together in the PCoA plots and were not significantly different in PERMANOVA and ANOSIM. We could culture few numbers of bacterial species including *Propionibacteria* and *Streptococci* from chicken embryo cecum samples after enrichment, while no bacteria were cultured from egg white samples. Although it is unknown how live bacteria are transferred to the embryo gut, evading the antimicrobial substances in the egg white, we assume that some bacteria in the egg white utilize their own strategies for survival, similar to *Salmonella* species, until the albumen is subsequently diluted with amniotic fluid, sub-embryonic fluid, or yolk during incubation and then absorbed into the embryo^39–42^. We failed to confirm the similarity between egg white, embryo, and the other sampling groups using ANOSIM. The SourceTracker2 test results showed the possibility that the over half of the egg white and embryo microbiota originated from the egg shell and that the maternal cloaca and oviduct microbiota contribute to the egg shell microbiota though the lack of sampling of environment near eggs limits this inference. Also, small sampling size limits this inference, therefore more study with large number of samples will be needed in a further study. Nevertheless, these results are somewhat consistent with the previous study of *Ding et al*.^26^, which suggested microbiota inheritance from mother hens to chicks based on a comparison of maternal feces and embryo microbiota. However, we included reproductive tract and egg white samples in our study and *Halomonas* species, picked as core bacteria of the maternal and embryo gastrointestinal microbiota by *Ding et al*.^26^, were only observed in one SPF egg white and three commercial Korean chicken oviduct samples in our study, with 0.02–0.04% relative abundance per sample. *Halomonas* species are frequently found in the contaminated saline, and the previous study did not include clean buffer only sample. Therefore, contamination of buffer by *Halomonas* species could be a reason of large abundance of *Halomonas* species in maternal feces and embryo microbiota in the previous study.

In this study, 21 taxa at genus level were observed commonly across the maternal cloaca and magnum, descendent egg white, egg shell, and embryo. These species made up >99% of the embryo microbiota, implying the nearly all of the chicken embryo microbiota is inherited from the maternal cloaca and/or oviduct. Eight genera were found in the magnum, egg white, and embryo intersection, but not in the cloaca and egg shell, and two genera were found in the cloaca, egg shell, egg white and, embryo intersection, but not in the magnum. The finding that no shared phylotype was found in the egg shell, egg white, and embryo intersection, also supports the hypothesis that bacteria in the cloaca and oviduct colonize the egg shell surface and egg white prior to other environmental bacteria. The colonized bacteria become the major component of embryonic microbiota; however, how transfer of the microbiota occurs remains unknown and still it is necessary to investigate the correlation between external environmental and embryonic microbiota. The relative abundance of the 21 common species was lowest in the magnum and the phylogenetic diversity of the magnum was higher than the other groups, suggesting that the oviduct microbiota has a effect on the cloaca microbiota and hence affects the egg shell, egg white, and embryo microbiota. In our other experiment, we could suggest that microbiota in hen cloaca consists of bacteria derived from rectum and oviduct. However, cause the results of this study are based on DNA sequence analysis, large number of those bacteria could be dead or not replicable in the egg white.

The presence of 110 phylotypes at the genus level found solely in the oviduct raises the question of their origin, although they only accounted for 2.4% of the bacterial population. The roles of these 110 genera and the other bacterial genera in the oviduct of host hens remain unknown.

To the best of our knowledge, this is the first study to examine mother hen oviduct, descendent egg, and chicken embryo microbiota using a metagenomic approach. Our results suggest that the bacterial genera diversity of the oviduct microbiota increases as chickens mature. The mature chicken reproductive tract microbiota showed different composition patterns compared to human and non-human mammals. Certain bacterial species present in the maternal oviduct microbiota comprised most of the embryo ceca bacterial population, though a small number of them could be cultured, indicating that the maternal oviduct microbiota could be partially transferred to offspring during egg formation. These findings suggest that egg white, normally considered as sterile, contains seed of bacteria that will become gut microbiota of embryo. These results could serve as the basis for studying the reproductive tract microbiota of other chicken breeds and different avian species. In addition, our results provide a useful reference for the analysis of microbiota changes in the reproductive tract of avian species with reproductive tract diseases or egg-transmitted pathogen infections.

## Methods

### Sampling of oviducts, eggs, and chicken embryos from Korean commercial breed chickens

Four fresh bodies of 32-weeks-old commercial Korean laying hens were donated by a local farm. The mucosal surface of each part of the oviduct (infundibulum, magnum, isthmus, and uterus) was aseptically scraped with the back of a scalpel blade. The exfoliated mucosa and mucus was collected in 1 mL of sterilized phosphate buffered saline (PBS).

### Sampling of oviducts, cloaca, eggs, and chicken embryos from SPF chickens

All experiments involving chickens were conducted according to the guidelines of the Institutional Animal Care and Use Committee (IACUC). The animal experiments were approved by the IACUC of Konkuk University (approval number: KU17103-1).

Twenty-five SPF hens were raised in isolation from the age of 14 weeks. The hens were fed with commercial chicken feed without any probiotics and antibiotics. Pre-lay oviduct samples (n = 8) were collected from 23 weeks-old hens. Artificial insemination was conducted every week while the hens were laying to produce fertilized eggs. The semen was donated from a local layer farm. Descendant eggs were collected from each hen and an area of at least 4 cm² on the egg shell surface was swabbed with a CLASSIQ Swab (COPAN, Murrieta, CA, USA), which was then suspended in 2 mL of PBS on the day of lay. In order to investigate possibility of transfer of environmental bacteria from egg shell to chicken embryo gut, we did not disinfect the egg shell and handled egg with sterilized instruments before swabbing. The cloaca of hens was swabbed with the CLASSIQ Swab and then suspended in 2 mL of PBS; 4 mL of egg white was collected from each egg using a syringe with a 17 G needle. Some of the fertilized eggs from each mother hen were incubated for 18 days and the cecum of the 18 day-old embryos was then collected and chopped using sterilized surgical instruments. The chopped ceca were transferred into 1 mL of sterilized PBS for DNA extraction. Oviduct samples (n = 16) were collected from 34 weeks-old laying hens. The magnum of the oviducts, collected from 23 weeks-old immature SPF hens and 34 weeks-old laying SPF hens, were scraped with the backside of a scalpel blade and suspended in 1 mL of PBS and stored at −20 °C until DNA extraction. In total, 24 oviduct, 14 egg white, 14 egg shell, 14 cloaca, and 14 chicken embryo cecum samples were used for DNA extraction and sequencing.

### DNA extraction and the IonTorrent sequencing

DNA was extracted from all PBS suspended samples using the DNeasy blood and tissue kit (Qiagen, Manchester, UK) according to the manufacturer’s instruction. DNA extraction from PBS used for sample preparation was conducted as negative control. The V2, V3, V4, V6-7, V8, and V9 regions of the 16S rRNA gene were amplified with the primer sets from the Ion 16S Metagenomics Kit (Thermo Fisher Scientific, MA, USA). Sequencing was conducted using the Ion S5 XL sequencer and Ion 530 chip.

### Sequence analysis

#### Sequence processing

QIIME2^43^ was used as the analysis pipeline and Greengenes^44^ (13_8 release) was used as a 16S rRNA sequence reference. The first 15 bases of all reads were trimmed to achieve a length of 150 bases and Phred quality score >15. The reads were denoised using dada2 and chimeric sequences were identified using the vsearch implemented in QIIME2 with a closed-reference algorithm. The sequences flagged as non-chimeras were retained. Sequence reads with >99% identity were clustered into a single OTU by vsearch^45^. OTUs with <10 copies were considered as artifacts and deleted. The OTUs were then classified using the naïve-Bayes classifier. To remove sequencing effort heterogeneity, samples were rarefied to 5,000 sequences. Of the 96 sample sequencing data set, two oviduct samples, which failed to yield 5,000 high quality (>15) reads, and one oviduct and one egg shell samples, showing too-different composition compared to other samples in the same location group, were excluded from analysis.

### Characterization of the oviduct, egg white, and embryo microbiota of commercial breed chickens

The relative taxa abundance of the groups is presented as a mean % value. Alpha diversity was measured using the Faith PD and Shannon indexes. Beta diversity plots were constructed to visualize the categorical partition of the samples using unweighted UniFrac^46^. To assess the association between microbial community and sampling location within the oviduct, egg, and embryo gut, pairwise PERMANOVA and ANOSIM analysis implemented in QIIME2 was performed on an unweighted UniFrac distance matrix of 63 samples. The significance of PERMANOVA was obtained by 999 permutation tests.

### Identification of bacterial taxa associated with sampling location

Differential abundance analysis by Analysis of Composition of Microbiomes (ANCOM) was used to identify significantly and differentially abundant genera in each sampling location.

### Ethics approval and informed consent

All experiments involving chickens were conducted according to the guidelines of the Institutional Animal Care and Use Committee (IACUC). The animal experiments were approved by the IACUC of Konkuk University (approval number: KU17103-1).

## Supplementary information

Supplementary file 1

Supplementary file 2

## Data Availability

The raw sequence reads from the metagenomic libraries were deposited in the NCBI Sequence Read Archive under BioProject accession number: PRJNA486876.
