# Supplementary material for: Characterization of microbial communities in the chicken oviduct and the origin of chicken embryo gut microbiota
Source: Sci Rep. 2019 May 2;9:6838. doi: 10.1038/s41598-019-43280-w (PMC6497628; doi:10.1038/s41598-019-43280-w)
Supplement: Supplementary file 1 [file 41598_2019_43280_MOESM1_ESM.pdf]

Characterization of microbial communities in the chicken oviduct and the origin of chicken embryo gut microbiota

Sangwon Lee[1], Tae-Min La[1], Hong-Jae Lee[1], In-Soo Choi[1], Chang-Seon Song[1], Seung-Yong Park[1], JoongBok Lee[1] and Sang-Won Lee[1]*

[1] College of Veterinary Medicine, Konkuk University, 120 Neungdong-ro, Gwangjin-gu, Seoul 05029, Republic of Korea

Supplementary File	Supplementary figure/table	Title of figure/table
Supplementary File 1	Figure S1	Comparison of microbial population in the oviducts of Korean commercial and SPF chickens.
	Figure S2	Rarefaction curve comparing Faith_pd values of SPF maternal hens' cloaca, oviduct, descendants' egg white, egg shell, and embryo.
	Figure S3	Shannon's diversity index of SPF maternal hens' cloaca, oviduct, descendants' egg white, egg shell, and embryo.
Supplementary File 2	Table S1	Relative abundance of bacteria in oviduct of Korean commercial breed hens.
	Table S2	Relative abundance of bacteria in SPF hen and descendant sample groups.
	Table S3	Relative abundance of core genera found in SPF hen and descendant sample groups.

*Supplementary File 2 is attached as separated file.

Supplementary File 1. Phylogenetic diversity and alpha diversity in hens, eggs and embryos

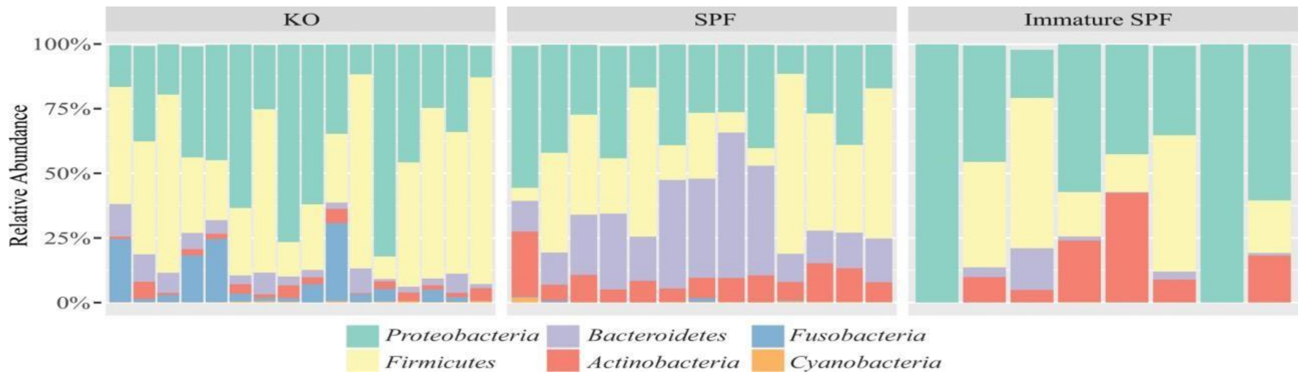

Figure S1. Comparison of microbial population in the oviducts of Korean commercial and SPF chickens.

Taxonomic diversity plot showing the relative abundance of taxa at the phylum level in each oviduct sample. KO: oviduct samples including infundibulum, isthmus, magnum and uterus from Korean commercial breed chickens; SPF: magnum samples from SPF breed chickens; Immature SPF: magnum samples from immature SPF breed chickens.

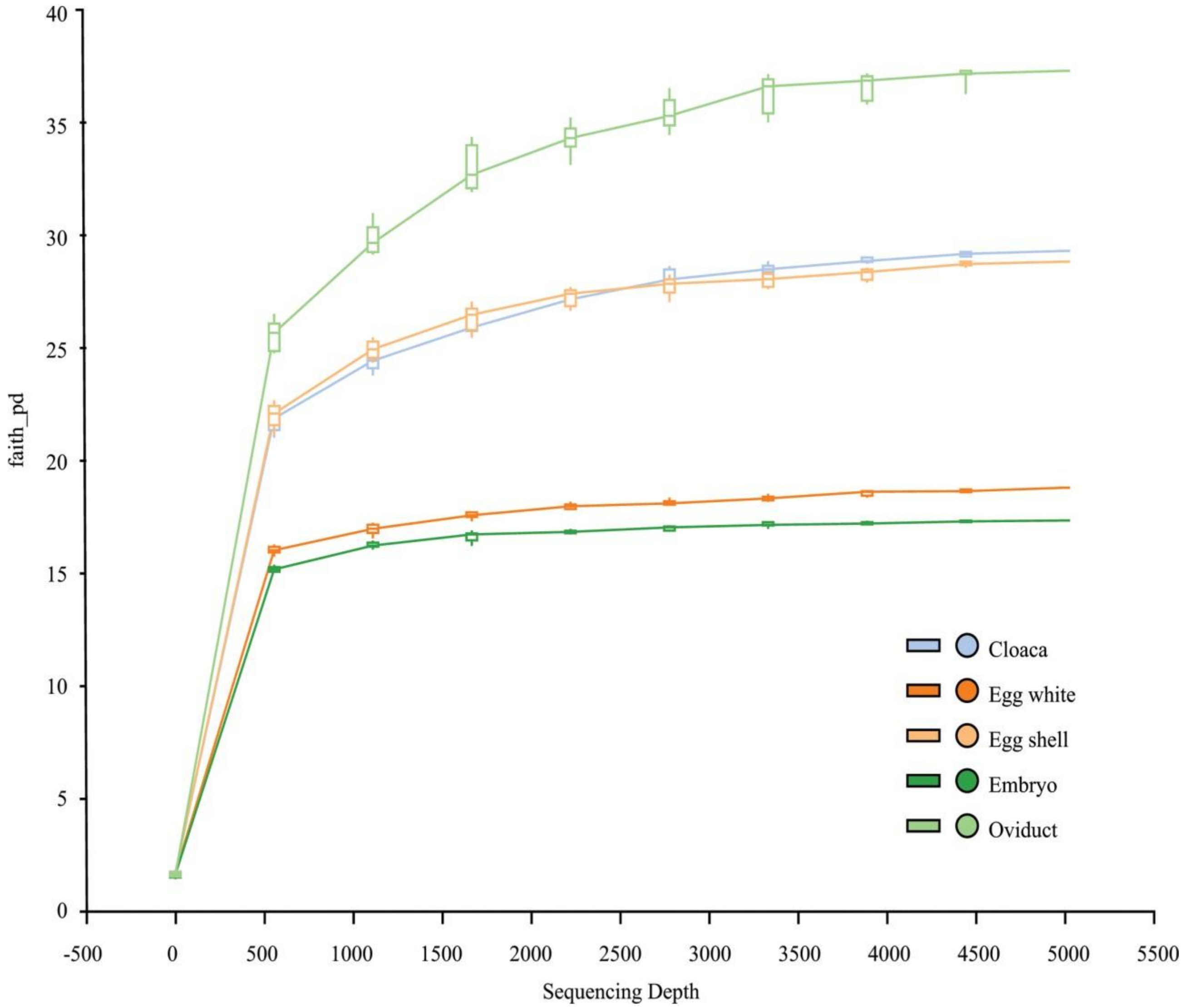

Figure S2. Rarefaction curve comparing Faith pd values of SPF maternal hens' cloaca, oviduct, descendants' egg white, egg shell, and embryo. Oviduct, SPF hen magnum; Cloaca, SPF hen cloaca; Egg shell, egg shell surface of eggs laid by SPF hens; Egg white, egg white of eggs laid by SPF hens; Embryo, cecum of 18-day-old chicken embryos laid by SPF hens.

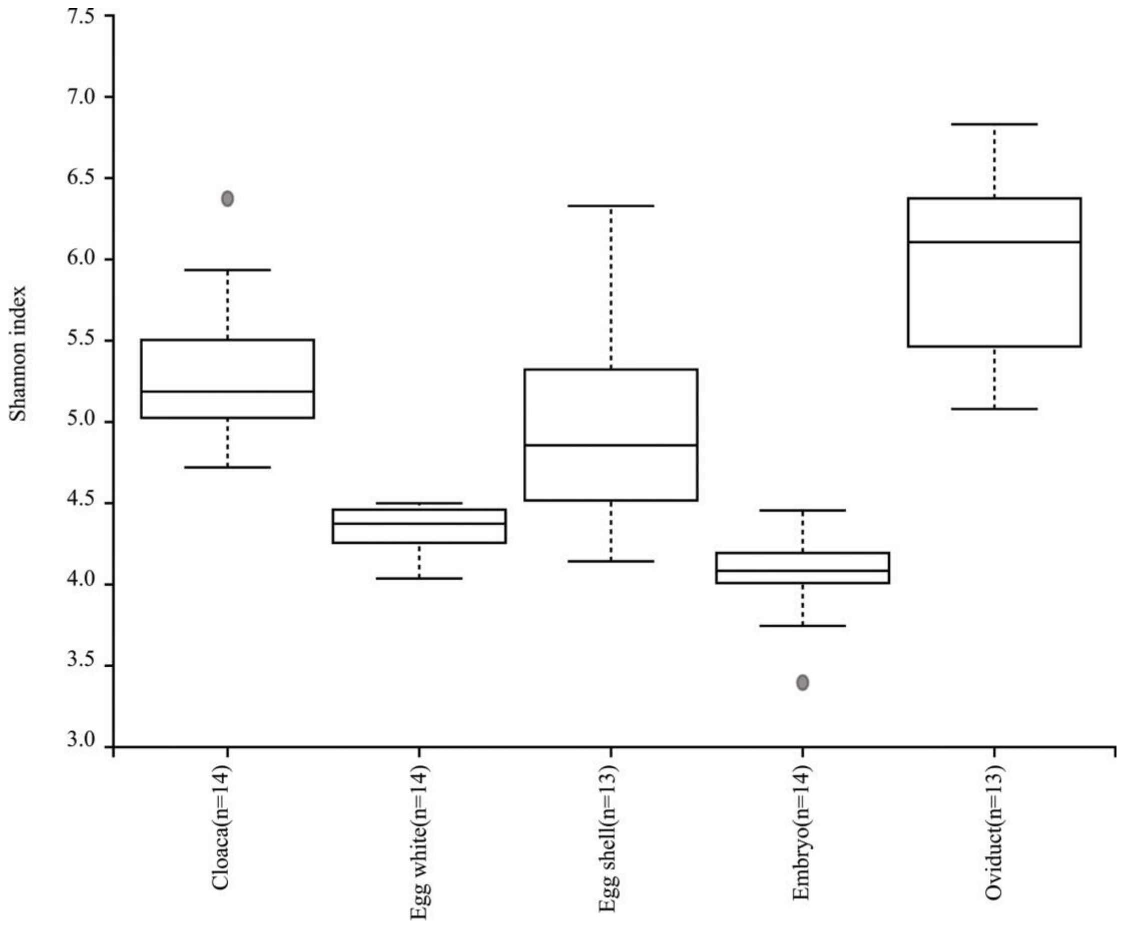

Figure S3. Shannon's diversity index of SPF maternal hens' cloaca, oviduct, descendants' egg white, egg shell, and embryo.

Oviduct, SPF hen magnum; Cloaca, SPF hen cloaca; Egg shell, egg shell surface of eggs laid by SPF hens; Egg white, egg white of eggs laid by SPF hens; Embryo, cecum of 18-day-old chicken embryos laid by SPF hens. n, the number of samples.
